# *Plasmodium falciparum* transmission based on merozoite surface protein 1 (*msp1*) and 2 (*msp2*) gene diversity and antibody responses in Ibadan, Nigeria

**DOI:** 10.1016/j.parepi.2024.e00366

**Published:** 2024-07-04

**Authors:** Tolulope A. Oyewole, Nurat O. Mohammed, Bright O. Osarenren, Muyideen K. Tijani, Kristina E.M. Persson, Mofolusho O. Falade

**Affiliations:** a Cellular Parasitology Programme, Cell Biology and Genetics units, Department of Zoology, University of Ibadan, Ibadan, Nigeria; b Division of Clinical Chemistry and Pharmacology, Department of Laboratory Medicine, Lund University, 22185 Lund, Sweden; c Clinical Chemistry and Pharmacology, Laboratory Medicine, Office for Medical Services, Region Skåne, 22185 Lund, Sweden; d Department of Biology, Transylvania University, KY, USA

**Keywords:** Malaria, *Plasmodium falciparum*, *msp1*, *msp2*, IgG, Antibody

## Abstract

**Background:**

Nigeria is a major contributor to the global malaria burden. The genetic diversity of malaria parasite populations as well as antibody responses of individuals in affected areas against antigens of the parasite can reveal the transmission intensity, a key information required to control the disease. This work was carried out to determine the allelic frequency of highly polymorphic *Plasmodium falciparum* genes and antibody responses against schizont crude antigens in an area of Ibadan, Nigeria.

**Materials and methods:**

Blood was collected from 147 individuals with symptoms suspected to be malaria. Malaria infection was determined using a rapid diagnostic test (RDT), and *msp1* and *msp2* were genotyped by a nested PCR method. In addition, levels of IgG directed against *P. falciparum* FCR3S1.2 schizont extract was measured in ELISA.

**Results:**

Approximately 25% (36/147) were positive for a *P. falciparum* infection in RDT, but only 32 of the positive samples were successfully genotyped. MAD20 was the most prevalent and K1 the least prevalent of the *msp1* alleles. For *msp2*, FC27 was more prevalent than 3D7. The mean multiplicities of infection (MOI) were 1.9 and 1.7 for *msp1* and *msp2*, respectively. IgG levels correlated positively with age, however there was no difference in median antibody levels between RDT-positive and RDT-negative individuals.

**Conclusion:**

Low MOI has before been correlated with low/intermediate transmission intensity, however, in this study, similar levels of *P. falciparum*-specific antibodies between infected and non-infected individuals point more towards a high level of exposure and a need for further measures to control the spread of malaria in this area.

## 1 Introduction

The burden imposed by malaria is enormous. In 2022, there were 249 million cases of malaria resulting into 608,000 deaths (WHO, 2023). *P. falciparum* is responsible for most of the fatal malaria cases worldwide, especially in sub-Sharan Africa. Both international and national strategies involving the use of insecticide-treated nets (ITN), long-lasting insecticide nets (LLIN), artemisinin combination therapy (ACT) and intermittent preventive treatment in pregnancy (IPTp) have ensured a reduction in the burden of malaria. In Nigeria, the deployment of these strategies has reduced the malaria prevalence from 42% in 2010 to 27% in 2015 (National Malaria Elimination Programme - NMEP/Nigeria et al., 2016). However, Nigeria alone is still responsible for 27% of cases of malaria globally (WHO, 2023).

Constant surveillance of malaria transmission intensity in endemic areas is important for proper evaluation of control strategies and planning. Depending on the techniques/methods utilised, surveillance could also reveal silent evolution going on in the parasite population, for example artemisinin resistance (Balikagala et al., 2021) or reduced immunity/upsurge in morbidly that usually follow after successful control strategies(Trape et al., 2011; Varela et al., 2020). Genetic diversity at polymorphic loci of the malaria parasites´ genome has been used as the basis for determining malaria transmission intensity, based on studies that demonstrated simple linear relationships between genetic diversity and transmission intensity (Anderson et al., 2000; Pumpaibool et al., 2009). In these studies, endemic areas in Africa had high genetic diversity while low/intermediate genetic diversity was recorded in low transmission areas such as in Thailand. However, inbreeding, choice of molecular technique used, infection by parasites of the same genotype, and choice of molecular markers can influence the determination of multiplicity of infection (MOI) and thus complicate the linear relationship between transmission intensity and genetic diversity of parasite isolates (Escalante et al., 2015; Zhong et al., 2018).

Genetic diversities of *msp1* and *msp2* have commonly been used to determine MOI of *P. falciparum* in affected areas across different geographical areas (Atroosh et al., 2011; Chen et al., 2018; Funwei et al., 2018; Jamil et al., 2021; Ogouyemi-Hounto et al., 2013; Singana et al., 2019). There are three allelic forms of *msp1*:RO33, K1 and MAD20 based on polymorphisms in the block 2 region (Miller et al., 1993; Tanabe et al., 1987). FC27 and 3D7 are the two main allelic forms of *msp2,* based on diversity of the regions flanking the central repeat sequences (Smythe et al., 1990, Smythe et al., 1991).

Serosurveillance that rely on the measurement of plasma antibodies directed against a broad spectrum of antigens is another useful tool for monitoring exposure and transmission in endemic areas, since antibodies are sensitive markers of exposure (Helb et al., 2015a; Stanisic et al., 2009), especially in low transmission areas. In this current study we determined *P. falciparum* transmission intensity in Lagelu local government area, Ibadan, Nigeria, by determining *msp1* and *msp2* allelic diversities in infected individuals. Antibody responses against schizont extract obtained from the FCR3S1.2 *P. falciparum* strain was also determined.

## 2 Materials and methods

### 2.1 Study site and sample collection

This study was carried out in Lagelu Local Government (LLG) area, which is located within the Ibadan metropolis, 145 km north of Lagos. This area is characterised by an alternation of two distinct seasons: a rainy season occurring from April to October in which malaria transmission is at its highest, followed by a dry season from November to March. This particular study was carried out from August 2019 to January 2020. Samples were collected from 147 individuals visiting five primary health care centres within the LLG; Monatan, Aiyegoro, Iyana-church, Alegongo and Olorunda. Only individuals presenting symptoms consistent with malaria (fever, headache, nausea and chills) were recruited for this study, after signing a consent form. Patients who had HIV/AIDs or other chronic diseases were exempted. 1–5 mL of blood was collected from each participant into EDTA tubes by venepuncture. Plasma was removed after centrifugation and stored at −20 °C alongside the pelleted cells. A drop of each of the blood samples was placed on RDT strips (Paracheck) to detect *P. falciparum*.

### 2.2 DNA extraction and *msp*1/*msp*2 genotyping

Genomic DNA was extracted from samples that were RDT positive (*n* = 36) using a Qiagen kit (QIAamp mini) following the manufacturer's instruction and stored at −20 °C until use. Block 2 of *msp1* and block 3 of *msp2* were amplified using a modified nested PCR technique described by Snounou et al. (1999). Briefly, specific primer pairs (Table S1) were used to amplify sequences covering the polymorphic regions of the two genes. The product of the primary reaction was used as template for five different second reactions (nested) to detect the K1, MAD20, RO33 (*msp1*), FCR27 and 3D7 (*msp2*) allelic variants.

The PCR amplification was performed on an Eppendorf thermal cycler (Eppendorf Master cycler AG22331) in a final volume of 25 μL. The cycling conditions for the primary PCR for both *msp1* and *msp2* were as follows: 95 °C for 5 mins for initial denaturation, 94 °C for 1 min for standby, 60 °C for 30 s for annealing; 72 °C for 2 mins for initial extension, 72 °C for 5 mins for extended elongation; except that the annealing temperature for *msp2* was 59.6 °C for 30 s. A total number of 35 cycles were performed for each reaction. 2.5 μL of primary PCR product was used as a DNA template in the secondary PCR, which had similar concentrations to the primary PCR. The cycling condition was the same for the secondary reaction as well but with annealing temperatures of 58 °C for MAD20 and K1, 60 °C for RO33, 51.3 °C for FC27 and 56.6 °C for 3D7, and for 30 s each. The amplified DNA fragments were detected on a 1.5% agarose gel.

### 2.3 Multiplicity of infection (MOI)

The mean MOI of the *msp1* and *msp2* genes was determined by calculating the sum total of different alleles at each locus for all samples and dividing by the total number of samples positive for each marker. Infections were also classified as single or multiclonal infections. Single infections had only one allele per locus while multiclonal infections were those with more than one allele in at least one locus out of all the loci genotyped.

### 2.4 Shizont extract preparation

Synchronized culture of FCR3S1.2 containing late trophozoites and schizonts was passed through MACs (Miltenyi) columns inserted into a magnet. The MACs column retained late trophozoites and schizonts, which were then eluted by removing the columns from the magnet and flushing the column with RPMI 1640 (Gibco) containing 0.5% BSA (Sigma Aldrich). The eluted parasites were pelleted by centrifugation at 2200 rpm for 3 min, washed x2 with PBS and finally resuspended in 100 μL of PBS. This was then subjected to four freeze-thaw cycles. Concentration of the extract was determined using a nanodrop spectrophotometer (Thermo Scientific).

### 2.5 Detection of IgG antibodies by ELISA

Maxisorp immunoplates were coated with 50 μL of FCR3S1.2 schizont extract (20 μg/mL) in PBS and incubated overnight at 4 °C. Plates were washed x3 with PBS/0.05% Tween 20 and then blocked with 100 μL/well of Blocker™ Casein (Thermo Fisher Scientific) for 2 h at 37 °C. Plates were washed again and 50 μL/well of plasma samples (diluted 1:50 in Blocker™ Casein) were added to the plates in duplicates and incubated at 37 °C for 1 h. Washing was repeated and 50 μL/well of alkaline phosphatase-conjugated goat anti-human IgG secondary antibody in Blocker™ Casein was added and plates incubated at 37 °C for 1 h. Plates were then washed and 50 μL/well of para-nitrophenol phosphate in ethanolamine buffer was added and colour developed in the dark for 30 min before absorbance was read at 410 nm. The diluent solution was used as the blank, and the absorbance value from it was subtracted from the absorbances of all the test samples. Only 95 samples were assayed due to small volumes of plasma caused by aliquots lost as a result of power failures.

### 2.6 Data analysis and management

Microsoft Excel version 16.72 (Microsoft Corporation, WA, USA) was used for data collation while SPSS version 22 (SPSS Inc., Chicago, IL, USA) and GraphPad Prism 9 (GraphPad Software, MA, USA) were used for analysis of the data. Median antibody levels for RDT positive and RDT negative individuals were compared using a Mann-Whitney test. Kruskal-Wallis was used to compare median antibody levels between individuals infected by different numbers of alleles.

### 2.7 Ethical approval

Ethical approval for this study was obtained from the Oyo State Ministry of Health ethical approval committee (AD13/479/1449). Permission was obtained from the Chief Matron of each of the individual health centres to carry out the research. Similarly, permission was obtained from each participant via a signed consent form before samples were collected. Participation was voluntary and refusal to participate did not attract any penalty with regards to the benefit of the study. The participants were also given the opportunity to ask questions before the commencement of the study.

## 3 Results

### 3.1 Demography of the study population

The study included 144 females (98%) and 3 males (2%). 56 (38.8%) of the female participants were pregnant. The level of involvement in intermittent preventive treatment in pregnancy (IPTp) among the pregnant participants was 73.2% (41/56). A total of 16 (10.9%), 27 (18.4%), 8 (5.4%), 40 (27.2%), and 56 (38.1%) samples were obtained from Aiyegoro, Iyana-church, Alegongo, Monatan and Olorundaa primary healthcare centres respectively. Most of the participants were adults (93.9%), but the age ranged from 2 to 79 years of age. In this study, 77 of the respondents (52.4%) indicated that they used anti-malarial medications without prescription whenever needed, and 71 respondents (48.3%) used insecticide treated mosquito nets.

### 3.2 Malaria positive individuals

Out of the 147 samples tested for malaria using the RDT, a total number of 36 samples (24.5%) were positive for *P. falciparum* antigens.

### 3.3 Frequency of *msp*1 and *msp*2 alleles

Only 32 samples out of the 36 RDT-positive samples were successfully genotyped. Out of the 32 isolates genotyped for *P. falciparum msp1*, 26 (81.3%), 21 (65.6%), and 15 samples (46.9%) were positive for the allelic families MAD20, RO33 and K1, respectively. For the *msp2* genotyping, 27 (84.4%) and 28 samples (87.5%) were positive for the allelic families 3D7 and FC27, respectively (Table 1). For one sampe, neither FC27 nor 3D7 could be detected (1/32).

**Table 1:** Proportion of each allelic family in the study population.

Allelic families	No positive by PCR	Proportion
*msp1*		
MAD20	26	81.3%
RO33	21	65.6%
K1	15	46.9%

*msp2*		
3D7	27	84.4%
FC27	28	87.5%

### 3.4 Multiplicity of infection

The mean MOI for *msp1* and *msp2* were 1.9 and 1.7, respectively. Based on the *msp1* alleles' distribution, 13 (40.6%), 8 (25%) and 11 (34.4%) of all genotyped samples were infected by one, two and three alleles, respectively.

### 3.5 Relationship between IgG antibody responses against FCR3S1.2 and age

**Fig. 1:**
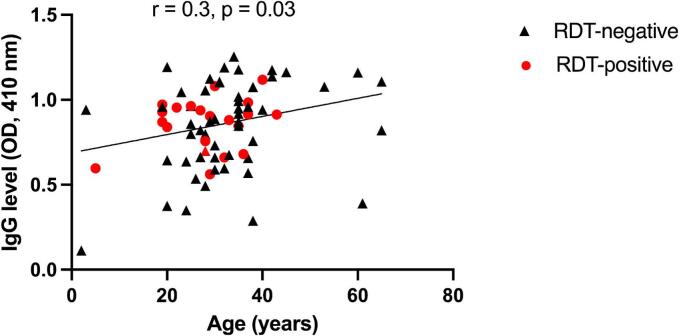
Spearman's correlation between IgG antibody levels against schizont extract and age (*r* = 0.3, *p* = 0.03).

**Fig. 2:**
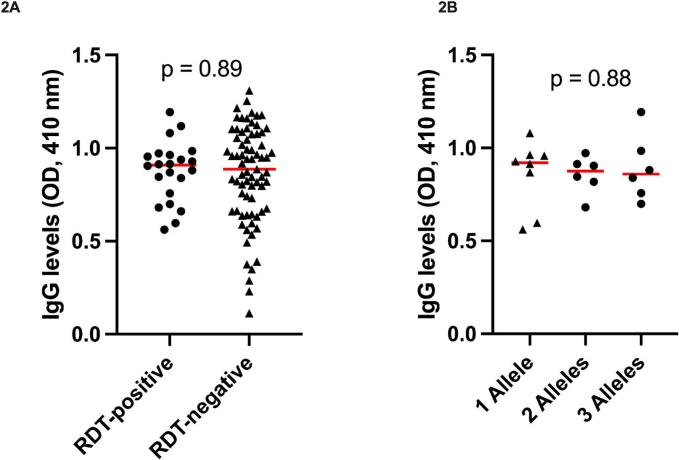
Comparison of median IgG antibody levels between RDT positive and RDT negative individuals, p = 0.89, (A); and between individuals infected by one, two and three *msp1* alleles, *p* = 0.9, (B).

There was a positive correlation between the levels of antibodies produced against schizont extract and age (*r* = 0.3, *p* = 0.03) (Fig. 1). We further compared antibody levels between RDT positive and RDT negative individuals, but there was no difference in antibody levels between these two groups (*p* = 0.89) (Fig. 2A). Furthermore, there was no difference in median antibody levels between individuals with mixed infections and those with single infections using Kruskal-Wallis test, based on the *msp*1 genotype (*p* = 0.88) (Fig. 2B).

## 4 Discussion

In this study we determined the genetic diversity of *P. falciparum* in individuals living in an urban area of Ibadan using the polymorphic regions of the *msp1* and *msp2* genes. *P. falciparum* was detected by RDT in only about 25% of individuals that presented with malaria symptoms. This unexpectedly low number of *P. falciparum* positive individuals among those with symptoms could be due to infections caused by other *Plasmodium* species, even though in this area *P.falciparum* is the main malaria parasite species in transmission (Mbama Ntabi et al., 2022). The symptoms could also be due to infections by other pathogens such as viruses (Chauhan et al., 2020; Tchuandom et al., 2019). The low RDT positivity could also be due to a prozone effect, although extremely high parasitemias would not be expected to be common. More positive individuals could also have been identified if PCR had been used since studies have shown that PCR could detect sub-microscopic parasitaemia out of reach of RDT and microscopic techniques (Kotepui et al., 2020; Mfuh et al., 2019).

The genetic diversity found here is comparatively low when compared with another study conducted in a similar setting in Ibadan (Funwei et al., 2018) and other parts of West Africa (Metoh et al., 2020). We found MAD20 to be the most prevalent of the *msp1* genes in our study area, contrary to the study done before in Ibadan (Funwei et al., 2018), where RO33 was found to be the most abundant. Some results from other parts of Southwestern Nigeria have found the K1 allele to be the most prevalent (Oboh et al., 2021; Olasehinde et al., 2012; Oyebola et al., 2014). In our study, K1 was the least prevalent, which is in agreement with an earlier study from Ibadan (Funwei et al., 2018). For the *msp2* gene, we found the FC27 allele to be more prevalent than 3D7, which is similar to one study (Olasehinde et al., 2012) but different from other studies (Funwei et al., 2018; Oyebola et al., 2014) conducted in the neighbouring areas of our study site. It is important to state that the prevalence of 3D7 (84.4%) and FC27 (87.5%) observed in our study were not significantly different from each other, and they were both common. In general, the distribution pattern of *msp1* and *msp2* alleles have before been shown to vary from location to location in different parts of the world (Ahmed Ismail et al., 2013; Chen et al., 2018; Jamil et al., 2021; Metoh et al., 2020; Ogouyemi-Hounto et al., 2013; Snounou et al., 1999). The mean MOI obtained in our study indicates an intermediate transmission intensity when compared with some of the studies cited earlier. This may be due to the impact of control measures that have been deployed over time, particularly the insecticide-treated nets(Bhatt et al., 2015), but it also indicates that control measures performed so far are not enough to control malaria infections in this area. Furthermore, the use of IPTp by some of the pregnant participants of this study may also have contributed to the relatively low transmission intensity recorded. Easy accessibility to anti-malarial medication, which is usually abused through self-medication (Omolase et al., 2011; Wegbom et al., 2021), is also a plausible explanation for low transmission of malaria parasites in the study population. It is possible that the use of a higher sample size in a similar study covering a bigger area may reveal a different level of genetic diversity from what is reported here.

**Supplementary Fig. 1:**
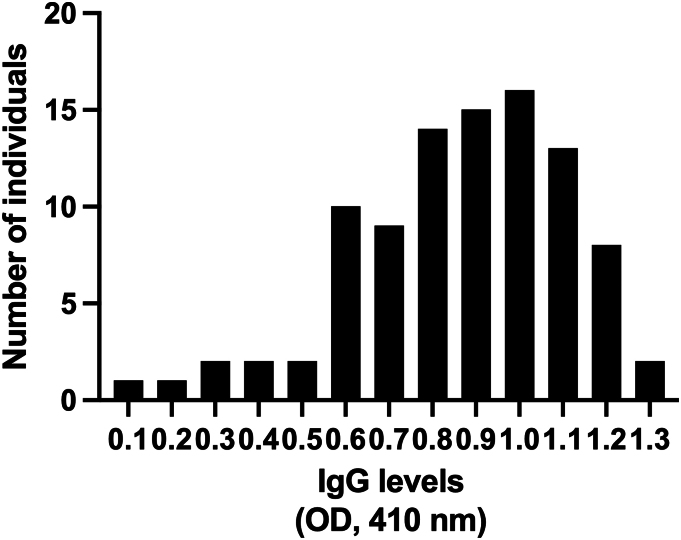
The distribution frequency of IgG antibody levels (absorbances) measured by ELISA. The distribution was not normal by D'Agostino and Pearson (*p* = 0.013), Anderson-Darling (*p* = 0.018) and Shapiro-Wilk (*p* = 0.008) tests, but slightly normal by Kolmogorov-Smirnov test (*p* = 0.056).

Measurement of antibody responses against schizont extract can be used to gain insights into the transmission intensity in a population (Stanisic et al., 2009; Varela et al., 2020). The moderate correlation between age and IgG antibody responses could be because the majority of participants here were adults who may have had similar experiences of malaria exposure and immunity. The comparable levels of IgG antibody levels seen between RDT positive and RDT negative individuals suggests that these two groups have had similar exposure in recent time and this might imply a high level of exposure/transmission in our study population. However, it is important to note that individuals with the lowest antibody levels were RDT negative. The levels of antibodies observed in RDT positive individuals were not surprising as parasitemic individuals often possess strong antibody responses against *P. falciparum* antigens (Greenhouse et al., 2011). Antibodies seen in RDT negative individuals may be lingering antibody responses to a recent infection that has been cleared by the immune system or through the use of conventional anti-malarial medications or herbs. The ability of antibodies to remain in the system long after the parasites have been cleared is a major advantage when levels of antibody responses against a broad-spectrum antigen is used as a proxy in determining exposure to pathogens. It is possible that the dilution factor used here was to high to detect meaningful differences between groups of highly immune individuals. However, higher or identical dilution factors had been used to detect IgG antibodies produced against asexual stage antigens in similar settings (Ahmed Ismail et al., 2014; Ngoundou-Landji et al., 2010; Omosun et al., 2005; Tijani et al., 2017). Futhermore, the dilution factor used in this current study was selected after prior testing and the distribution of the absorbance values (Supplementary Fig. 1) clearly shows that the dilution factor was able to differenciate between specific IgG antibodies of different levels.Schizont extract is composed of soluble antigens expressed at the schizont stage and antibodies produced against them have been found to be predictive of recent malaria infections (Cook et al., 2010; Ondigo et al., 2020). Generally, the acquisition and maintenance of *P. falciparum*-specific antibodies are significantly affected by age, antigen and exposure intensity (Akpogheneta et al., 2008; Stanisic et al., 2015; Tijani et al., 2017) and estimated half-life of these antibodies range from a few weeks to about 80 years (Helb et al., 2015b; Ondigo et al., 2014). The affinity of *P. falciparum*-specific antibodies has also been shown to be affected by the same factors (Tijani et al., 2018). Similar levels of antibodies between individuals with multiple infections and those with single infections reinforces the existence of a relatively high exposure to *P. falciparum* in our study area. One of the limitations of this study is the low sample size, which was due to inherent challenges during the recruitment stage of this work. There is a possibility that a higher sample size in a similar study could show higher genetic diversity than what was reported here. Another limitation is the low resolution of our detection facility, which could not detect small differences in fragment sizes of the alleles. Use of better detection systems could also increase the observed genetic diversity of the allelic families beyond what we have demonstrated.

In conclusion, we have shown here that *P. falciparum* is still being transmitted in our study population. The genotyping of *msp1*/*msp2* has before been shown to be reliable in ascertaining the diversity of *P. falciparum* species and thus a useful tool in evaluating effectiveness of control strategies in endemic areas. Low MOI as found here has before been correlated with low/intermediate transmission intensity, however, in this study, similar levels of *P. falciparum*-specific antibodies between infected and non-infected individuals point more towards a high level of exposure. The inclusion of antibody measurements against broad spectrum antigens will also in future be useful for providing more comprehensive information about transmission intensity and evaluation of control strategies in affected areas.

The following are the supplementary data related to this article.

Supplementary material 1 *msp*1 and *msp*2 primary and secondary primer sequences. Supplementary material 1

## Funding

This work received support from TWAS and ALF (10.13039/501100003252Region Skåne/Lund University), SUS fonder, O. E. och Edla Johanssons stiftelse, Stiftelsen Sigurd och Elsa Goljes Minne and Alfred Österlunds stiftelse.

## CRediT authorship contribution statement

**Tolulope A. Oyewole:** Methodology, Writing – original draft. **Nurat O. Mohammed:** Methodology. **Bright O. Osarenren:** Methodology. **Muyideen K. Tijani:** Conceptualization, Methodology, Writing – original draft. **Kristina E.M. Persson:** Conceptualization. **Mofolusho O. Falade:** Conceptualization.

## Declaration of competing interest

Authors declare no conflict of interest.
