# Supplementary material for: Plasmodium falciparum transmission based on merozoite surface protein 1 (msp1) and 2 (msp2) gene diversity and antibody responses in Ibadan, Nigeria
Source: Parasite Epidemiol Control. 2024 Jul 4;26:e00366. doi: 10.1016/j.parepi.2024.e00366 (PMC11294720; doi:10.1016/j.parepi.2024.e00366)
Supplement: Supplementary material 1 msp1 and msp2 primary and secondary primer sequences. [file mmc2.docx]

Table S1: *msp*1 and *msp*2 primary and secondary primer sequences.

|  | | Primary PCR |  | |
| --- | --- | --- | --- | --- |
| Locus | | **Primer** | **Primer Sequence** | |
| *msp*1 | | *msp1*-1 | 5’ -CTAGAAGCTTTAGAAGATGCAGTATTG- 3’ | |
|  | | *msp1*-2 | 5’ -CTTAAATAGTATTCTAATTCAAGTGGATCA- 3’ | |
| *msp2* | | *msp2-1* | 5’-ATG AAG GTA ATT AAA ACA TTG TCT ATT ATA-3’ | |
|  | | *msp2-2* | 5’-ACA TTC ATA AAC AAT GCT TAT AAT ATG AGT-3’ | |
| Secondary PCR | | | | |
| Locus | **Primer** | | | **Primer Sequence** |
| *msp1* | MAD20-1 | | | 5’ -GCTTGCATCAGCTGGAGGGCTTGCACCAGA- 3’ |
|  | MAD20-2 | | | 5’ -AAATGAAGAAGAAATTACTACAAAAGGTGC- 3’ |
|  | R033-1 | | | 5’ -CATCTGAAGGATTTGCAGCACCTGGAGATC- 3’ |
|  | RO33-2 | | | 5’ -TAAAGGATGGAGCAAATACTCAAGTTGTTG- 3’ |
|  | K1-1 | | | 5’ -ATCTGAAGGATTTGTACGTCTTGAATTACC- 3’ |
|  | K1-2 | | | 5’ -AAATGAAGGAACAAGTGGAACAGCTGTTAC- 3’ |
| *msp2* | 3D7-1 | | | 5’-GCA GAA AGT AAG CCT TCT ACT GGT GCT-3’ |
|  | 3D7-2 | | | 5’-GAT TTG TTT CGG CAT TAT TAT GA -3’ |
|  | FC27-1 | | | 5’-GCA AAT GAA GGT TCT AAT ACT AAT AG-3’ |
|  | FC27-2 | | | 5’-GCT TTG GGT CCT TCT TCA GTT GAT TC-3’ |
